# Nutrition and Physical Activity in Aging, Obesity, and Cancer

**DOI:** 10.1111/j.1749-6632.2012.06735.x

**Published:** 2012-10-10

**Authors:** Jun Nishihira

**Affiliations:** Department of Medical Management and Informatics, Hokkaido Information UniversityEbestu, Hokkaido, Japan

**Keywords:** β-(1–3)-glucan, Crohn’s disease, Dectin-1, inflammatory bowel disease, macrophage migration inhibitory factor, ulcerative colitis

## Abstract

Macrophage migration inhibitory factor (MIF) is a unique protein that participates in inflammation, immune responses, and cell growth. An array of *in vitro* and *in vivo* experiments has demonstrated that MIF is profoundly involved in the pathogenesis of acute and chronic inflammatory disorders, such as inflammatory bowel disease (IBD). Blockade of MIF bioactivities by either neutralizing anti-MIF antibodies or antagonists prevents inflammatory cytokine cascade, which strongly suggests that an anti-MIF therapeutic strategy is feasible for treatment of IBD. Recently, we developed a new therapeutic approach for IBD by administration of antisense MIF oligonucleotides in conjugation with schizophyllan (SPG), a member of the glucan family. SPG specifically binds Dectin-1 expressed in antigen-presenting cells (APCs), and the antisense MIF/SPG complex is incorporated into the cells. In *in vivo* experiments of colitis models in mice, we found that intraperitoneal administration of the complex ameliorated the clinical signs of colitis and improved the histological scores. This novel therapy designed to knock down the MIF production in APCs is expected to be clinically applicable for the treatment of IBD.

## MIF as a proinflammatory cytokine, immunomodulator, and growth factor

Macrophage migration inhibitory factor (MIF) was originally discovered as a soluble mediator induced by a lymphoid cell–antigen interaction that is relevant to delayed-type hypersensitivity.^1^ MIF exerts a variety of biological functions, including proinflammatory actions, enhancement of immunological reactions, and tumorigenicity.^2^ An array of reports has demonstrated that MIF is expressed in a variety of cells other than immune cells, such as epithelial and endothelial cells. These findings strongly suggest that MIF is involved in various pathophysiological states beyond the immune system. As a proinflammatory cytokine, MIF is released from monocytes/macrophages in response to various stimuli, such as endotoxin and ultraviolet. Since the expression of inflammatory cytokines such as tumor necrosis factor (TNF)-α, interleukin (IL)-1, and IL-6 is markedly suppressed by anti-MIF antibodies,^3^ MIF is considered to have potential for promoting the expression of inflammatory cytokines. Taken together, these results show that MIF is ubiquitously expressed in both types of immune cells, for example, macrophages and epithelial cells, and functions as a proinflammatory cytokine and immunomodulator.

Moreover, several reports have suggested that MIF plays additional roles as a growth factor-like molecule in the events of tumor cell growth and wound healing. We reported that MIF plays a critical role in tumor growth and angiogenesis, as assessed by *in vivo* and *in vitro* studies using colon cancer cells.^4^ In brief, murine colon cancer cells (cell line colon-26) were inoculated subcutaneously into the right flank of BALB/c mice, and the volume of the resulting tumors was drastically reduced by administration of anti-MIF antibody. Furthermore, tumor-associated angiogenesis examined by a Millipore chamber filled with colon-26 cells in the subcutaneous fascia of the flank of mice was significantly suppressed by the administration of anti-MIF antibodies. As for the wound-healing process, we demonstrated that MIF mRNA was upregulated, and that anti-MIF antibody treatment significantly delayed the process of wound repair.^5^ According to these findings, it is conceivable that MIF promotes the growth of both tumor tissues and nontumor tissues in the event of wound repair.

## Molecular characteristics of MIF

With respect to the molecular characteristics of MIF, a number of studies have been published. After the determination of the complementary DNA (cDNA) sequence of MIF,^6^ the tissue distribution and a variety of the biological functions of MIF were reported. We isolated rat MIF cDNA, which contains an open reading frame of 345 base pairs that encodes 115 amino acid residues.^7^ The nucleotide sequence homologies of the rat MIF cDNA coding region to the corresponding human, mouse, and chicken sequences were 89.4%, 97.1%, and 73.3%, respectively. The MIF genomic nucleotide sequences of humans and other species have been cloned and their structures were found to have three exons separated by two short introns.^8^ The human *MIF* gene contains multiple Sp1 sites and a cAMP response element (CRE); however, the precise regulatory mechanism at the transcriptional level remains to be elucidated.

The human MIF gene is located on chromosome 22q11.23, which was linked to an accumulation of abdominal subcutaneous fat in caucasians in a genome-wide linkage scan.^9^ Two polymorphisms are known to exist in the promoter region of the MIF gene, a tetranucleotide CATT repeat located at position –794 (–794[CATT]_5–8_)^10^ and a single-nucleotide G/C substitution located at position –173 (–173G/C).^11^ We revealed that MIF –794[CATT]_5–8_ was associated with obesity in a Japanese population, while another polymorphism in the MIF promoter region, –173G/C, was not associated with obesity.^12^ It is of interest that MIF secretion from adipocytes is correlated with body mass index (BMI). MIF may function as a direct regulator of energy metabolism, and may affect adiposity as a proinflammatory cytokine in the manner of TNF-α. These findings suggest that MIF contributes to obesity via the regulation of inflammatory cytokine production in adipose tissues.

Following molecular cloning of the rat MIF gene, we crystallized the MIF rat protein and solved its three-dimensional structure, revealing that it has a homotrimeric form.^13^ Each monomer consists of two β/α/β motifs aligned in quasi-twofold symmetry. The domain consists of four-stranded mixed β-sheet and two antiparallel α-helices. The structure–function relationship made it clear that MIF possessed a number of molecular functions as isomerase activity, for example, tautomerase and oxidoreductase activities.^14^ In detail, MIF has an amino acid sequence homologous with d-dopachrome tautomerase, and an unexpected protein structural similarity with two bacterial isomerases, 5-carboxylmethy-2-hydroxymuconate isomerase and 4-oxalocrotonate tautomerase. Based on the similarities of the amino acid sequences and tertiary structures, we revealed that MIF possessed isomerase activities by molecular-based enzymatic analyses. Currently, the physiological significance of this activity is under investigation.

## The role of MIF in inflammatory bowel disease

Inflammatory bowel diseases (IBDs) such as Crohn's disease and ulcerative colitis (UC) are characterized by relapsing and chronic inflammation in the intestine, but their pathogenesis has not been fully elucidated. Studies on the pathogenesis and exacerbation of IBD have tended to focus on the imbalance of the proinflammatory cytokine cascade. The association between MIF and IBD has been extensively investigated because of the high expression of MIF in infiltrated macrophages and colonic epithelial cells. de Yong *et al*. demonstrated that the expression of *MIF* was increased in a model of experimental colitis induced by the transfer of CD45RB high, and that blockade of MIF with anti-MIF antibody reduced the severity of colitis.^15^

**Figure 1:**
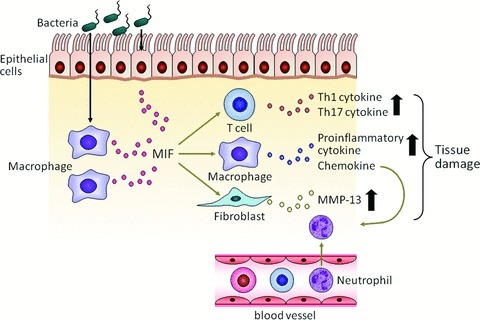
The hypothesized role of MIF in IBD. MIF would be released from immune cells such as macrophages in response to endotoxin in the event of colitis. In this figure, we show a model in which bacterial endotoxin invades colonic epithelial cells, which stimulates the release of MIF from macrophages in the colon tissue. Extracellularly released MIF would stimulate release of inflammatory cytokines such as TNF-α and trigger metalloprotease expression, leading to inflammation and tissue damage. In addition, MIF would potentiate the recruitment of neutrophils to the inflammatory site, exacerbating inflammation and tissue damage.

We demonstrated that *MIF*-deficient mice showed much less inflammation than wild-type mice.^16^ We assessed clinical signs, including diarrhea and rectal bleeding as well as histological scores of colitis when mice were given 3% or 10% dextran sulfate sodium (DSS). In all of these cases, few or no histological features of colitis were observed in *MIF*-deficient mice, and infiltration of neutrophils and macrophages was much less frequent. Considering these findings together, we hypothesize that MIF stimulates the accumulation of innate immune cells such as neutrophils and macrophages in the colon of mice with colitis (Fig. 1).

Since matrix metalloproteinase (MMP) is overexpressed in systemic and local inflammation in IBD and in experimental colitis, the protease is thought to be important in the development of colitis in cases of IBD.^17^ We demonstrated that the expression of *MIF* was increased in DSS-induced colitis in mice. In this model, neutralization of MIF with an anti-MIF polyclonal antibody suppressed the disease activity of colitis, suppressing the production of Th1-type cytokines and MMP-13 in the colon.^18^ MMP is thought to mainly mediate tissue degradation and remodeling. As for the signal transduction of MIF, expression of MMP in response to MIF was investigated using human synovial fibroblasts, which showed that MIF-induced MMP-1 and MMP-3 expression via a tyrosine kinase-, protein kinase C-, and AP-1–dependent pathway.^19^

It has been reported that the MIF protein was significantly increased in the sera of patients with Crohn's disease and those with UC.^20^ In the same study, MIF was shown to markedly enhance the production of IL-8 in dendritic cells obtained from patients with UC compared with non-UC patients.^21^ As for the G-to-C transition at position –173 of the *MIF* gene, this site became functionally active in the presence of C through the creation of an activator protein 4 transcription factor binding site.^22^ In the Japanese population, on the other hand, no significant difference in genotype distribution of the *MIF* gene was found between UC patients and healthy controls. Nonetheless, the frequency of the C/C genotype was significantly higher in patients with the pancolitis type of UC compared with other types of colitis in UC. Thus, the *MIF*–173 polymorphism may be related to the extent of the lesion in patients with UC in a Japanese population.

## Drug delivery system to Dectin-1–expressing immune cells

Schizophyllan (SPG) is a polysaccharide designated as a member of the β-(1–3)-glucan family that forms a triple helix in neutral solution. In alkaline solution, it is denatured and adopts a single-chain formation. When an alkaline solution of SPG is neutralized, the single chain reverts to its original triple helix through hydrophobic and hydrogen bond interaction. During this physicochemical interaction, two main-chain glucoses of β-(1–3)-glucans and one oligonucleotide (ODN) base form a stoichiometric complex, and by exploiting this complex, an SPG-based drug delivery system was designed to deliver functional ODNs to targeted cells.^23^ Dectin-1 was identified as a pathogen pattern recognition receptor that binds β-glucans such as SPG and is the major β-glucan receptor on APCs, for example, macrophages and dendritic cells.^24^ The complex, therefore, is presumably recognized by Dectin-1 on APCs, and ODNs are taken up by these cells.

## Effect of antisense MIF/SPG complex on DSS-induced colitis

Dectin-1 expression was significantly increased in the colon tissues from DSS-treated mice compared with samples from normal mice. Concurrently, MIF expression was clearly observed in DSS-treated tissues and infiltrated macrophages. In this context, it is conceivable that the DSS-induced colitis model is appropriate for investigation of the effectiveness of the antisense MIF/SPG complex in the treatment of colitis. Accordingly, we used the DSS-induced colitis model to carry out the following experiments.

From the data available to date, it is strongly suggested that MIF is a key factor in the pathogenesis of IBD, and the control of MIF expression would be a potential therapeutic approach. Based on this underlying notion, we used the antisense ODN sequence to suppress MIF, as the antisense ODN has a (dA)40 tail that induces the formation of a complex with SPG. In our experiment, the antisense MIF/SPG complex (200 μg/kg) was repeatedly administered (days 0, 3, 7, and 10) by intraperitoneal injection, and the disease activity index was evaluated on day 14.^25^ The administration of the antisense MIF/SPG complex led to significant protection against DSS-induced colitis, as evaluated by clinical signs and histopathological markers, including weight loss and colon shortening.

In the same study, we also performed endoscopic examinations and histological analysis of sections of the colon. Briefly, mucosal injury and inflammation were improved in the antisense-treated mice, and the histological scores were significantly reduced in the antisense-treatment group. In parallel, the serum MIF concentration was significantly inhibited by the antisense MIF/SPG complex. Furthermore, the MIF, IL-1β, and IL-6 levels produced in mesenteric lymph nodes and colon tissues were significantly decreased by the treatment. Therefore, it is concluded that the antisense MIF/SPG complex effectively inhibited MIF production from Dectin-1–positive cells such as macrophages, thereby attenuating intestinal inflammation. At present, we have been investigating possible reductions in the frequency and dose of complex injections.

Based on the antisense therapy data available to date, viral infections, cancers, inflammatory disorders, and genetic disorders have been targeted; however, these antisense therapeutic methods have not become very popular because of the instability of ODN, which is subject to rapid degradation by deoxyribonucleases. To overcome this drawback, enhanced stability of antisense MIF, ODN against deoxyribonucleases was successfully achieved by forming a triple helix with 1 molecule of antisense ODN and 2 molecules of SPG. Accordingly, the antisense MIF/SPG complex is effectively and specifically delivered to macrophages and dendritic cells expressing Dectin-1 on their membranous surface. As described above, this therapeutic method is theoretically designed using a molecular basis approach and would be advantageous for clinical use with high specificity targeting the diseased lesions of IBD.

## Perspectives

In this report, we have provided an overview of the molecular characteristics and biological aspects of MIF, particularly with respect to colitis, and discussed therapeutic approaches for targeting MIF for IBD. The administration of the antisense MIF/SPG complex significantly ameliorated colitis by suppressing MIF production without causing any adverse effects in a mouse model. Our study suggested that this delivery system targeting MIF would be a candidate treatment for IBD.

It has been reported that the expression of MIF was increased in colon cancerous lesions.^4^ Thus, an anti-MIF strategy for IBD treatment is advantageous not only for suppression of intestinal inflammation, but also for prevention of the colon cancer often seen at the late stage of IBD. In this context, antisense MIF/SPG complex therapy is clinically meaningful for prevention of IBD and colon cancer. Currently, a colonic route (enema) and oral administration of antisense MIF/SPG complex are under investigation. We have already seen hints of the effectiveness of these treatments, and further experiments are underway to more thoroughly investigate their efficacy against not only IBD but also rheumatoid arthritis and other chronic inflammatory disorders.
